# Wound Dressings Coated with Silver Nanoparticles and Essential Oils for The Management of Wound Infections

**DOI:** 10.3390/ma13071682

**Published:** 2020-04-03

**Authors:** Bogdan Stefan Vasile, Alexandra Catalina Birca, Mihaela Carmen Musat, Alina Maria Holban

**Affiliations:** 1 National Research Center for Micro and Nanomaterials, University Politehnica of Bucharest, Splaiul Independenţei Street, No. 313, 060042 Bucharest, Romania; ada_birca@yahoo.com; 2 Department of Science and Engineering of Oxide Materials and nanomaterials, Faculty of Applied Chemistry and Materials Science, University Politehnica of Bucharest, Polizu Street, No. 1-7, 060042 Bucharest, Romania; 3 Faculty of Engineering in Foreign Language, University Politehnica of Bucharest, Romania, Splaiul Independenţei Street, No. 313, 060042 Bucharest, Romania; mihaelamusat6@yahoo.com; 4 Department of Microbiology and Immunology, Faculty of Biology, University of Bucharest, Aleea Portocalelor, No. 1-3, 050663 Bucharest, Romania; alina_m_h@yahoo.com

**Keywords:** dip coating, silver nanoparticles, essential oils, antimicrobial

## Abstract

Infection represents one of the major risk factors in persistent and difficult to treat wounds. This study focuses on developing antimicrobial wound dressings coated with silver nanoparticles, sodium alginate and different essential oils, to avoid wound infection and biofilm formation. The design of the wound dressings was done by the dip coating method. The characteristics of the developed materials were analysed by physicochemical (FT-IR, XRD, SEM, TEM) and biological (antimicrobial tests) approaches. The results demonstrated uniform silver nanoparticle formation on the substrate, and the developed nanomodified dressings were proven to have increased antimicrobial and antibiofilm potential. The developed wound dressings based on silver nanoparticles, sodium alginate and essential oils have real potential in treating infections, and can be investigated as an efficient alternative to antibiotics and topical preparations for wound management.

## 1. Introduction

Infection represents one of the main risk factors in wound healing. When the skin function based on the barrier activity against bacterial invasion is lost, a large amount of exudate emerges at the affected site, which, together with the body temperature, provides an ideal place for bacterial growth. [1,2,3,4,5,6].

The most common Gram-positive bacteria that can appear at wound sites is *Staphylococcus aureus*, which can cause a wide range of infections. These bacteria can lead to significant infections, from the localized affected skin to life-threatening circumstances such as bacteraemia and even pneumonia. Another concern refers to the ability of this opportunistic pathogen to cause hospital-acquired and antibiotic-resistant infections. In many countries, the management of Methicillin-resistant *S. aureus* (MRSA) represents a difficult task in health care facilities [7,8,9,10,11]. Along with *Staphylococcus* species, Gram-negative bacteria (such as *Escherichia coli* and *Pseudomonas aeruginosa*), but also yeasts (ex. *Candida albicans*) could colonize wounds and produce persistent infections [12]. A major concern in infected wounds is the formation of biofilms, which are multicellular communities of microbial species that manifest particular behaviour and increased tolerance to high amounts of antimicrobial drugs and also to the host’s immune response [13].

Recently, there has been an increased interest in developing innovative wound dressings which are able to facilitate healing and avoid wound infection [12,14]. Biopolymers, i.e., naturally occurring proteins and polysaccharides with architectural resemblance to organic tissue obtained through appropriate processing (e.g., nanofibers, spongelike hydrogels, composites etc.), were successfully integrated into the design of dressings and coatings. Several functional biomaterials were identified and implemented for promising wound healing applications, with a reduced impact on the environment [15].

Sodium alginate is a natural polymer which can be found in the field of wound healing. Numerous biomedical applications are currently investigating this polymer for the development of controlled release drug delivery systems, as an extracellular matrix material for biological studies and scaffolds for tissue engineering in general. However, sodium alginate also has some drawbacks depending on its application, as compared to synthetic polymers, such as low thermal stability, high hydrophilicity and poor mechanical properties. In this respect, studies have led to the development of composite materials with alginate and other materials intended to improve the properties of this polymer [16,17,18].

In recent years, the field of nanostructured materials technology has been the object of major interest among scientists, since different types of metallic (gold, silver) or oxide (copper, zinc, titanium, magnesium) nanomaterials have attractive properties and functionalities. Silver nanoparticles have been shown to be very efficient in the development of bioactive dressings with applications in anti-infectious therapy [19,20], due to their demonstrated antimicrobial effect against bacteria, viruses and other microorganisms [21]. Due to their low toxicity to human cells, silver nanoparticles are suitable candidates for use as antibacterial agents in wound dressings [22,23]. 

Essential oils (EOs) are obtained from plants (flowers, leaves, roots, buds or bark) as oily extracts using methods such as extraction, fermentation or steam distillation. Some EOs are known to contain natural antimicrobial compounds due to their strong antimicrobial and antioxidant characteristics [24,25]. In this study, we selected three of the most actively antimicrobial (i.e., clove EO is active against numerous Gram-positive, Gram-negative and fungi strains [26,27]) and widely used bioactive EOs (i.e., niaouli and mandarin EOs are used in the cosmetic, pharmaceutic and food industries [28,29]). Mandarin oil is mostly used in skin treatment because it has a role in reducing fluid retention and stimulating skin circulation, and may reduce the appearance of scars and wrinkles [30,31]. Niaouli oil is also known for its strong antibacterial effect. This oil stimulates the immune system by increasing the white blood cell count and antibodies leading to it being known as an effective immunostimulant essential oil [32].

Clove oil possesses special antimicrobial properties. This oil is one of the most effective medicinal herbs against a wide range of yeasts and fungi, as well as against Gram-positive and Gram-negative bacteria [33,34].

In this paper, silver nanoparticles and sodium alginate, together with mandarin, niaouli and clove EOs, were used to demonstrate the properties of functional silver nanoparticles in improving the antimicrobial activity of classic dressings.

## 2. Materials and Methods 

### 2.1. Synthesis of Silver Nanoparticles

For the synthesis of silver nanoparticles, two solutions were prepared: one obtained by dissolving 0.5 g of silver nitrate (AgNO_3_) in 100 mL of distilled water, and the other, the reductant, by dissolving 1 g of D-glucose and 4 g of sodium hydroxide (NaOH) in 100 mL of distilled water, under continuous stirring at 80 °C. After the preparation of the two solutions, they were mixed by dripping the reductant solution into the silver nitrate solution, a process that modified the colour. The final product was homogenous in nature and was centrifuged, washed, dried for 48 h and mortared for further analysis [35].

### 2.2. Synthesis of The Modified Wound Dressings 

Traditional textile wound dressings were purchased from a local supplier and sectioned in four lots of 14 parts, with a size of 1 cm × 1 cm. In order to obtain the desired wound dressings, we chose to make four solutions (one of silver nanoparticles used as a control, and three other silver nanoparticles solutions, each of which was functionalized with different essential oils, i.e., mandarin, niaouli and clove). 

To improve the coverage and properties of the dressings, we chose to make a sodium alginate solution by dissolving 1 g alginate (Alg) in 100 mL of distilled water under continuous stirring until homogenization occurred.

The control solution was obtained using the same method as the one mentioned above. After that, one lot of wound dressings was introduced in the silver nanoparticles solution under magnetic stirring for two minutes, until the nanoparticles were incorporated in the wound dressing. Then, the wound dressings were removed from the solution and dropped in the sodium alginate solution for two minutes. Finally, the wound dressings were removed from the sodium alginate solution and deposited on a glass support to dry.

The solutions functionalized with essential oils were obtained using the same method, with the only difference being that in the reductant solution, we added 500 µL of each of the three essential oils. Four lots of wound dressings were produced, including one lot of wound dressings with silver nanoparticles and alginate layer, a second lot with silver nanoparticles plus mandarin oil and alginate layer, a third with silver nanoparticles plus niaouli oil and alginate layer, and a fourth with silver nanoparticles plus clove oil and alginate layer.

### 2.3. Physico-Chemical Characterization

X-ray diffraction (XRD) studies were carried out using a Malvern PANalytical Empyrean (Almelo, The Netherlands) equipped with a hybrid monochromator 2 x Ge 220 on the incident side and a parallel plate collimator mounted on the PIXcel 3D detector on the diffracted side. Grazing Incidence X-ray Diffraction (GIXRD) measurements were performed at room temperature, with an incidence angle ω = 0.5° for Bragg angle values of 2θ between 10° and 80°, using Cu Kα radiation with λ = 1.5406 Å (40 mA and 45 kV).

The morphology of the samples was analysed using a Quanta Inspect F FEG (field emission gun) scanning electron microscope (SEM) with 1.2 nm resolution equipped with an energy-dispersive X-ray (EDX) analyzer with a resolution of 133 eV at MnKα (Thermo Fisher (former FEI) (Hillsboro, OR, USA).

The high-resolution transmission electron microscope (TEM) images of the samples were obtained on finely powdered samples using a 80–200 KV Titan Themis high-resolution TEM from (Thermo Fisher (former FEI) (Hillsboro, OR, USA). The microscope was operated in transmission mode at 200 kV.

### 2.4. Antimicrobial Evaluation

Antimicrobial tests were performed in vitro by using three clinically relevant and model microbial species: Gram-negative (*Escherichia coli ATCC 25922*, Gram-positive: *Staphylococcus aureus* ATCC 23235 and the yeast strain *Candida albicans ATCC 10231*. The antimicrobial properties of the developed materials were analysed by qualitative (i.e., evaluation of growth inhibition zone) and quantitative methods (i.e., growth of planktonic and attached cultures). For the evaluation of growth, the inhibition zone fragments (1 cm × 1 cm) of the developed materials were sterilized by UV exposure for 30 min. Sterile materials (from each sample) were aseptically placed on nutritive agar plates previously inoculated with 0.5 McFarland (1.5 × 10^8^ colony forming units/mL) microbial suspensions prepared from the tested microbial strains. The inoculation of microbial strains was performed according to the disc-diffusion antibiogram assay using a sterile swab. The inoculated Petri dishes containing the tested materials were incubated at 37 °C for 24 h to allow microbial development. After incubation, growth inhibition was evaluated. After the incubation time, the growth inhibition zone developed around and under the materials was measured.

To assess the impact of the coatings on microbial growth in both free-floating (planktonic) and attached cultures, selected microbial strains were grown in nutritive broth in the presence of the coatings. One fragment of sterile material from each sample was placed in a six-well sterile plate. Then, 2 mL of nutritive broth was added into the plates and they were inoculated with 20 μL each of the prepared microbial 0.5 McFarland suspensions. These plates were incubated for 24 h at 37 °C to allow microbial growth and biofilm formation. After incubation, the absorbance of planktonic cultures developed in the presence of the coatings was measured using a spectrophotometer at a set absorbance of 600 nm.

In order to assess the ability of the microbial cells to attach and develop biofilms after 24 h of incubation, the wound dressings from the previously prepared six-well plates were washed with sterile saline water and introduced in sterile Eppendorf tubes in 1 mL sterile saline water. The washed coatings containing attached cells were further vigorously vortexed to detach the attached cells, and then the resulting suspensions were diluted and inoculated on agar plates to perform viable count analysis and calculate colony forming units (CFU)/mL values. Statistical significance was analysed by Student’s T-test. Values of P lower than 0.05 were considered statistically significant.

## 3. Results and Discussion 

### 3.1. Silver Nanoparticles Characterization 

#### 3.1.1. X-ray Diffraction (XRD)

**Figure 1:**
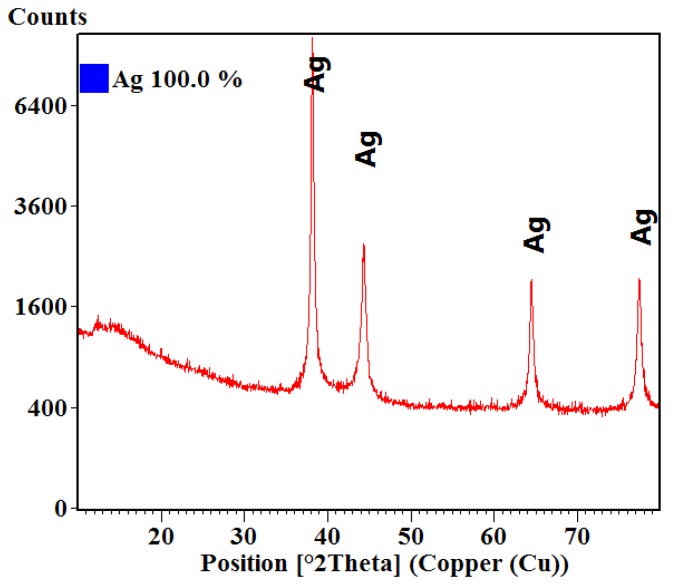
X-ray diffractogram for silver nanoparticles powder.

Figure 1 represents the XRD diffractogram obtained on the silver nanoparticles powder, where the crystallinity and the phase of interest are analysed.

It was observed that the interferences of the powder corresponded to pure phase cubic silver. The spectra presented diffraction peaks for the defined Miller indices planes (111), (200), (220), (311), in accordance with the American Society for Testing and Materials (ASTM) sheet for silver (ICDD PDF4+ 00-004-0783). The degree of crystallinity of the sample is evident by the intensity of the diffraction lines. The crystallite size was also calculated using the Scherrer formula; the obtained average crystallite size was 21.60 nm.

#### 3.1.2. Scanning Electron Microscopy (SEM)

**Figure 2:**
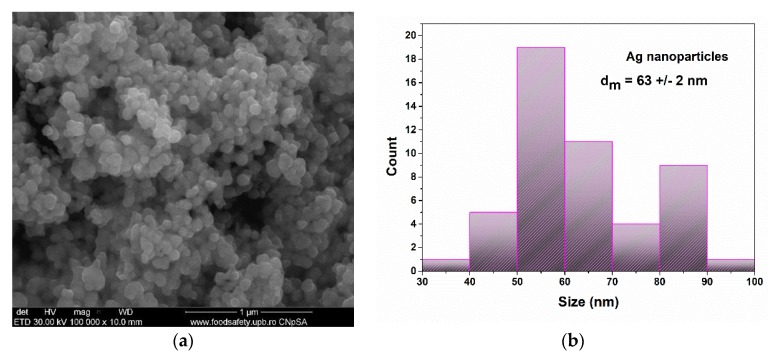
SEM analysis (**a**) and histogram (**b**) of the silver nanoparticle powder.

In order to investigate the sample morphology and the particles sizes, a SEM analysis was performed. In Figure 2, the SEM images are presented for the silver nanoparticle powder and the measured particle size distribution. 

In Figure 2, the structure and size of the nanoparticles are analysed. The nanoparticles are in agglomerated powder. The particles presented almost spherical shapes, which are known for their antimicrobial activity. As seen in the particle size distribution graph, the mean particle size was 63 +/− 2 nm. The nanoparticles were also distributed bimodally with a maxima at around 55 nm and 85 nm.

#### 3.1.3. Transmission Electron Microscopy (TEM)

**Figure 3:**
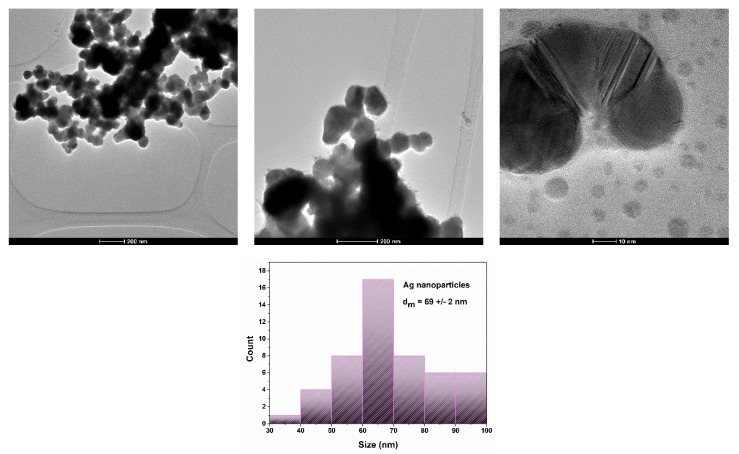
TEM analysis of silver nanoparticles powder.

To better observe the morphology and crystallinity of the nanoparticles, a TEM analysis was performed. The images are shown in Figure 3.

TEM images for the silver nanoparticle powder reveal that the particles are composed of soft agglomerates with a connection between all the independent particles. The morphology of the nanoparticles is characterized by their polyhedral shape. The average particle is 69 +/− 2 nm. The crystallite size calculated from XRD analysis (21.603 nm) highlights the polycrystalline nature of the particles.

### 3.2. Wound Dressings Characterization

#### 3.2.1. Scanning Electron Microscopy (SEM)

To evaluate the distribution of the silver nanoparticles in the commercial wound dressing, the samples with nanoparticles, sodium alginate and different essential oils were analysed with the SEM equipment. In the Figures below SEM images of the obtained modified commercial dressings are shown.

**Figure 4:**
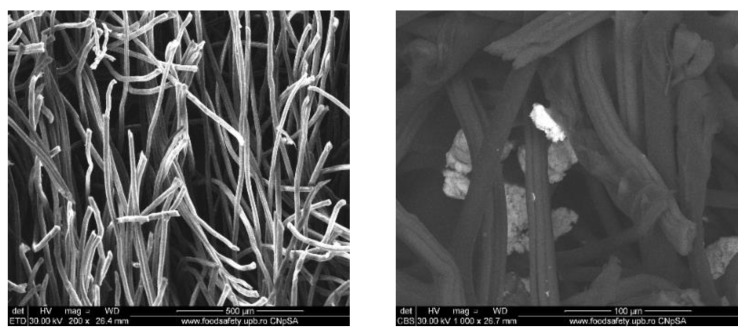
SEM images of wound dressings with silver nanoparticles and sodium alginate. **Left**: global image, and **right**: detailed image.

The images obtained on the wound dressing covered with sodium alginate and dipped into the Ag nanoparticle solution show that the coating of the dressing with nanoparticles formed from nanoparticle agglomerates and some individual distributed nanoparticles, as shown in Figure 4.

**Figure 5:**
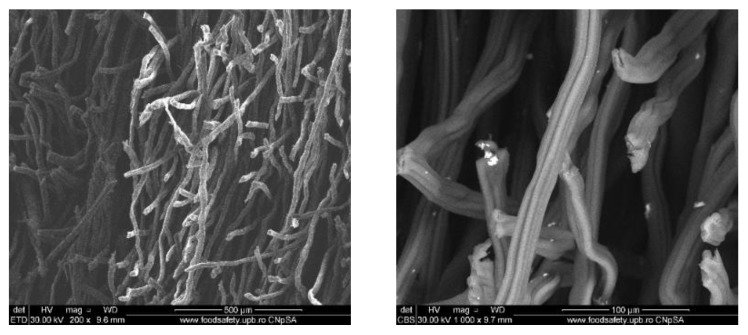
SEM images of wound dressings with silver nanoparticles, sodium alginate and mandarin oil. **Left**: global image, and **right**: detailed image.

For the samples also coated with mandarin oil, we can see that the oil itself has a big influence in nanoparticle distribution on the surface of the dressing (Figure 5). Thus, the nanoparticles are still present in agglomerated form but with smaller dimensions and with improved coverage on the surface of the fibers, as well as being more homogenous. 

**Figure 6:**
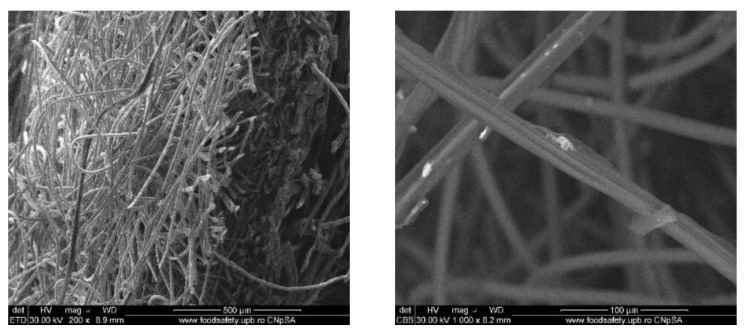
SEM images of wound dressings with silver nanoparticles, sodium alginate and niaouli oil. **Left**: global image, and **right**: detailed image.

Figure 6 shows the SEM images of the wound dressing with silver nanoparticles and niaouli oil. For these samples, the addition of the essential oil resluted in improved formation and distribution of the silver nanoparticles. Moreover, the oil properties influence the nanoparticles’ behaviour so that they adhere to the dressing fibers. 

**Figure 7:**
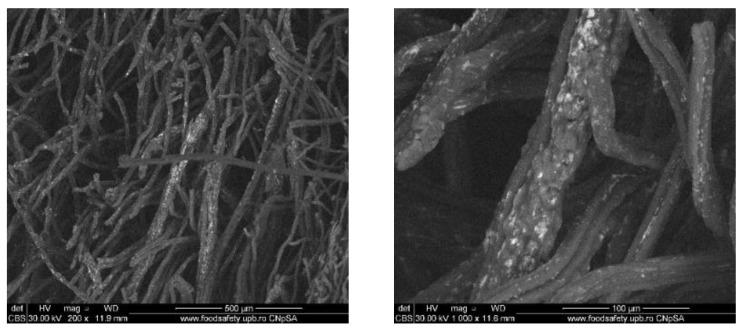
SEM images of wound dressings with silver nanoparticles, sodium alginate and cloves oil. **Left**: global image, and **right**: detailed image.

In Figure 7, the SEM images for the fourth sample which contain clove oil are shown. In the SEM images, the commercial wound dressings with silver nanoparticles, alginate and clove oil show a clear and obvious accumulation of silver nanoparticles on the surface and between the dressing fibers. Moreover, the nanoparticles are more homogenous and significant in quantity compared to the other samples. The clove oil exhibits best improves the distribution of the silver nanoparticles on the modified dressing. 

#### 3.2.2. Antimicrobial Activity-Evaluation of Growth Inhibition Zone

**Figure 8:**
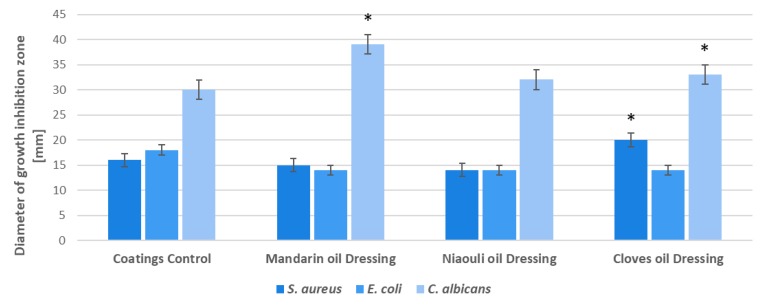
Evaluation of growth inhibition zone for *S. aureus*, *E. coli* and *C. albicans* in the presence of the developed wound coatings: Coatings Control—dressing modified with silver NPs, Mandarin oil Dressing—dressing modified with silver NPs and mandarin EO, Niaouli oil Dressing—dressing modified with silver NPs and niaouli EO, Clove oil Dressing—dressing modified with silver NPs and clove EO. * *p* < 0.05 (control coatings vs. NPs and EOs coated samples for each strain).

Our data revealed that after 24 h incubation at 37 °C, all the used materials exhibited diverse inhibition zones, with diameters ranging from 14–20 mm for *S. aureus*, 14–18 mm for *E. coli* and 30–39 mm for *Candida albicans* (Figure 8). The highest inhibition zone was observed for the *C. albicans* tested strain, where zone inhibition reached 39 mm for dressings coated with mandarin essential oil (EO) functionalized silver nanoparticles (NPs). In all the tested strains, the coatings containing nanoparticles functionalized with mandarin and clove EOs manifested the highest antimicrobial impact (Figure 8).

#### 3.2.3. Antimicrobial Activity-Growth of Planktonic and Attached Microbial Cultures

**Figure 9:**
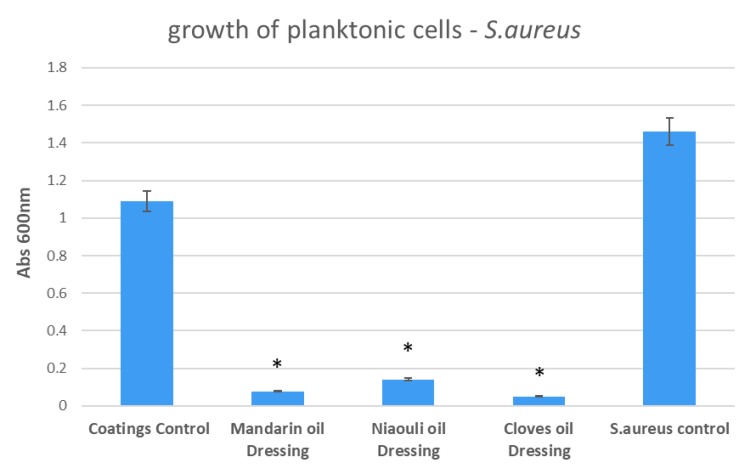
Growth of planktonic cultures of *S. aureus* in the presence of wound dressings coated with EOs functionalized silver nanoparticles (NPs). Coatings Control—dressing modified with silver NPs, Mandarin oil Dressing—dressing modified with silver NPs and mandarin EO, Niaouli oil Dressing—dressing modified with silver NPs and niaouli EO, Clove oil Dressing—dressing modified with silver NPs and clove EO. * *p* < 0.05 (control coatings vs. NPs and EOs coated samples for each strain).

The growth of planktonic culture was different in the presence of the designed nanocoatings, depending on the utilized essential oil and the tested microbial cells. In *S. aureus*, the most significant growth inhibition was achieved in the presence of cloves and mandarin EOs functionalized silver nanoparticles (Figure 9). However, niaouli EO functionalized silver nanoparticles (NPs) also exhibited significant inhibition of planktonic culture growth in *S.aureus* (i.e., more than four fold).

**Figure 10:**
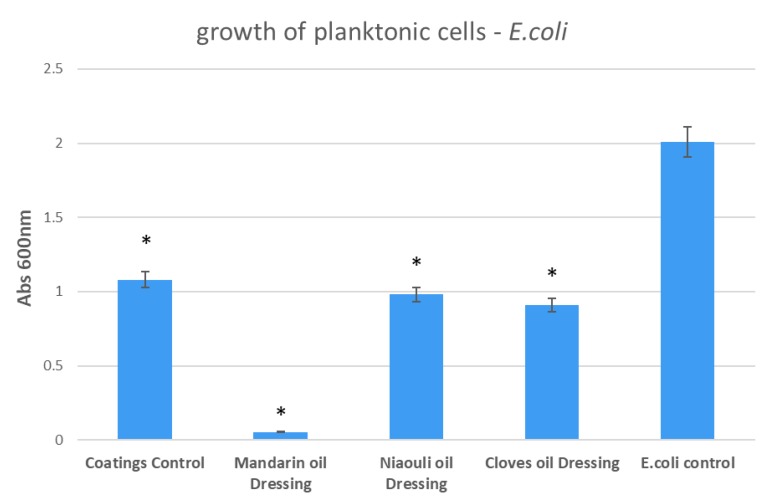
Growth of planktonic cultures of *E. coli* in the presence of wound dressings coated with EOs functionalized silver nanoparticles (NPs). Coatings Control—dressing modified with silver NPs, Mandarin oil Dressing—dressing modified with silver NPs and mandarin EO, Niaouli oil Dressing—dressing modified with silver NPs and niaouli EO, Clove oil Dressing—dressing modified with silver NPs and clove EO. * *p* < 0.05 (control coatings vs. NPs and EOs coated samples for each strain).

In the case of *E. coli*, the most significant growth inhibition was achieved in the presence of mandarin EO functionalized silver NPs, with the values obtained for niaouli and clove EOs being similar to those obtained for the plain silver NPs (Figure 10). 

**Figure 11:**
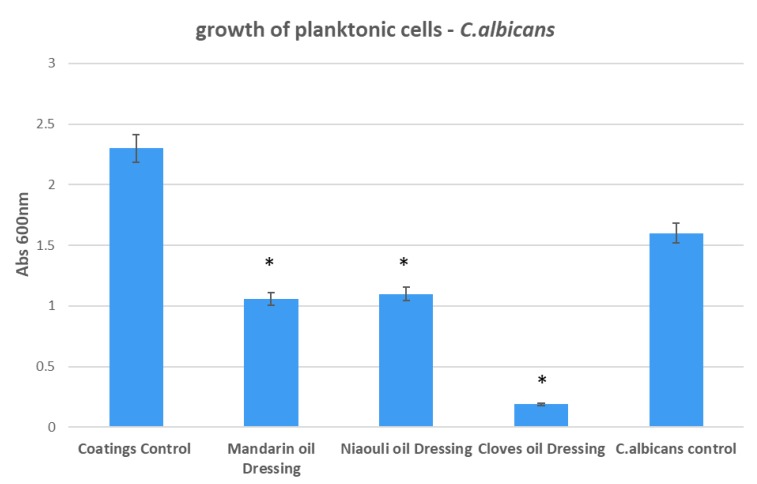
Growth of planktonic cultures of *C. albicans* in the presence of wound dressings coated with EOs functionalized silver nanoparticles (NPs). Coatings Control—dressing modified with silver NPs, Mandarin oil Dressing—dressing modified with silver NPs and mandarin EO, Niaouli oil Dressing—dressing modified with silver NPs and niaouli EO, Cloves oil Dressing—dressing modified with silver NPs and clove EO. * *p* < 0.05 (control coatings vs. NPs and EOs coated samples for each strain).

In the case of *C. albicans*, the most significant growth inhibition was achieved in the presence of clove EO functionalized silver NPs. However, mandarin and niaouli EOs functionalized NPs also manifested a high inhibition of planktonic cultures (Figure 11).

**Figure 12:**
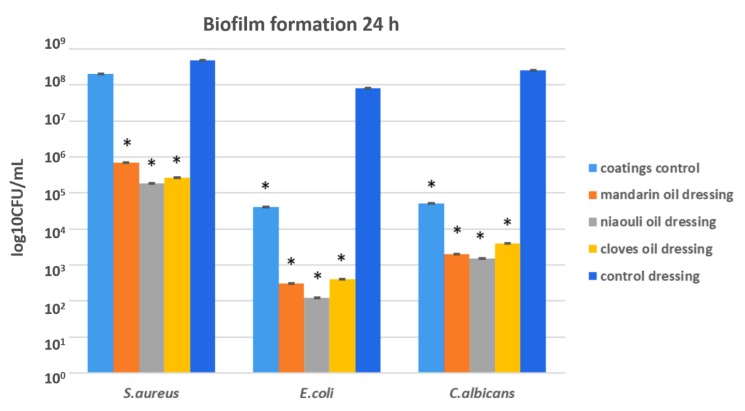
Biofilm formation in the presence of tested nanocoatings coatings control = microbial cells attached to the coatings control-dressing modified with silver NPs. Mandarin oil dressing = microbial cells attached to the mandarin dressing modified with silver NPs and mandarin EO. Niaouli oil dressing = microbial cells attached to the niaouli dressing modified with silver NPs and niaouli EO. Clove dressing = microbial cells attached to the cloves dressing modified with silver NPs and clove EO. Control dressing = microbial cells attached to the commercial wound dressing (containing silver NPs and EO free). * *p* < 0.05 (control coatings vs. NPs and EO coated samples for each strain).

In order to evaluate the ability of microbial cells to develop biofilms on the developed materials, cells were incubated for 24 h in the presence of *S. aureus*, *E.coli* and *C. albicans*, after which the correlated results were recorded, as shown in the graph below. The results demonstrated that all the tested nanocoated wound dressings had an inhibitory effect against microbial attachment by *S. aureus*. Compared with the nanoparticle free control, the most significant attachment inhibition was observed in the case of clove and niaouli nanoparticle coatings (Figure 12). All EO functionalized silver nanoparticle coated wound dressings have an enhanced antibiofilm effect, especially the niaouli containing dressing, on the Gram-negative, *E. coli* and the yeast tested strain, *C. albicans*. This suggests that niaouli EO is more efficient against microbial attachment and biofilm formation, while mandarin and clove EOs are more efficient at diminishing microbial growth in planktonic, free floating cells.

Moreover, from the SEM images obtained for the designed wound dressings, it can be seen that the clove oil influences the distribution of silver nanoparticles. A correlation can be made between the presence of clove oil along with the abundance of silver NPs on the dressing and the results after the antimicrobial activity. We can say that there is a synergistic effect between clove oil and silver nanoparticles, as highlighted by the evaluation of the growth inhibition zone and growth of planktonic cells, where clove oil was the most effective, compared to the others used. Overall, the SEM results show that the presence of niaouli oil improves the silver nanoparticles distribution on the commercial dressing. In this sense, the effect of niaouli oil dressing against biofilm formation is increased.

## 4. Conclusions

Natural products such as plant-derived extracts and essential oils could be useful in the therapy and prevention of infectious diseases, especially in the context of increasing antibiotic resistance rates. Moreover, nanotechnology represents an efficient way to improve the activity of such bioactive compounds and potentiate their antimicrobial activity. In this paper, we evaluated the potential of silver NPs functionalized with EOs to inhibit microbial colonization and biofilm formation on nanocoated wound dressings. The use of natural compounds and materials such as alginate and essential oils (mandarin, clove and niaouli) together with silver NPs have permitted the development of improved wound dressings which could be efficiently utilized for the management of wounds, by avoiding infection without the use of antibiotics and antiseptic topic products.
